# Screening for serine/threonine kinases phosphorylating Stat5

**DOI:** 10.1186/1471-2210-11-S2-A33

**Published:** 2011-09-05

**Authors:** Angelika Berger, Andrea Hölbl, Wolfgang Warsch, Veronika Sexl

**Affiliations:** 1 Institute of Pharmacology and Toxicology, University of Veterinary Medicine Vienna, 1210 Vienna, Austria; 2 Institute of Pharmacology, Center of Physiology and Pharmacology, Medical University of Vienna, 1090 Vienna, Austria

## Background

Stat transcription factors are highly conserved key regulators of cellular processes such as proliferation, differentiation, growth and apoptosis. Stats have been frequently found to be deregulated in cancer. One example is the constitutive activation of Stat5 in chronic myelogenous leukaemia (CML). The disease is caused by a chromosomal translocation resulting in the expression of the Bcr/Abl fusion kinase. The transcription factor Stat5 is required for Bcr/Abl-induced leukaemic initiation and disease progression. Furthermore, serine phosphorylation of a constitutive active form of Stat5 is crucial for haematopoietic transformation. These findings put Stat5 into the spotlight of new therapeutic tactics. Here, we screen for kinases phosphorylating Stat5 by employing a cell viability-based screening approach.

## Methods

Stable leukemic cell lines expressing variants of the *bcr/abl* oncogene and different levels of the Stat5 protein were screened for the induction of apoptosis with purchased kinase inhibitor libraries. Additionally, cells over-expressing phospho-mimetic mutants were included in the screening and served as controls for specificity.

## Results

We showed that serine phosphorylation of Stat5 is required to prevent apoptosis in Bcr/Abl-dependent cell lines. Therefore, we used a cell viability-based screening assay showing that 42 of 300 kinase inhibitors induced apoptosis in Bcr/Abl-dependent cell lines. Predominately, those hit compounds were inhibiting members of the CMGC kinase family, which are primarily proline-directed serine/threonine kinases. Furthermore, cell lines with endogenous levels of Stat5 and over-expressing wild-type Stat5 were compared in an additional round of screening. The resulting 6 hit compounds, which inhibit CDKs, GSK3, PKC and p38 MAP kinase, are currently being validated by Western blot analysis with phospho-Stat5 specific antisera.

## Conclusions

These findings point towards the importance of CMGC kinase family members for survival of Bcr/Abl-dependent leukaemic cell lines.

